# Associations of physical activity, fitness, and body composition with heart rate variability–based indicators of stress and recovery on workdays: a cross-sectional study

**DOI:** 10.1186/1745-6673-9-16

**Published:** 2014-04-18

**Authors:** Tiina Teisala, Sara Mutikainen, Asko Tolvanen, Mirva Rottensteiner, Tuija Leskinen, Jaakko Kaprio, Marjukka Kolehmainen, Heikki Rusko, Urho M Kujala

**Affiliations:** 1 Department of Health Sciences, University of Jyväskylä, P.O. Box 35, Jyväskylä FIN-40014, Finland; 2 Methodology Centre for Human Sciences, Faculty of Social Sciences, University of Jyväskylä, P.O. Box 35 (Y 33), Jyväskylä FI-40014, Finland; 3 Turku PET Centre, University of Turku, Kiinamyllynkatu 4-8, Turku FIN-20520, Finland; 4 Hjelt Institute, Department of Public Health, University of Helsinki, P.O. Box 41, Helsinki FI-00014, Finland; 5 Institute of Public Health and Clinical Nutrition, Clinical Nutrition, University of Eastern Finland, Kuopio Campus, P.O. Box 1627, Joensuu FIN-70211, Finland; 6 Department of Biology of Physical Activity, University of Jyväskylä, P.O. Box 35, Jyväskylä FIN-40014, Finland; 7 Department of Mental Health and Substance Abuse Services, National Institute for Health and Welfare, P.O. Box 30, Helsinki FI-00300, Finland; 8 Institute for Molecular Medicine (FIMM), University of Helsinki, P.O. Box 20, Helsinki FI-00014, Finland

**Keywords:** Body composition, Body fat percentage, BMI, Cardiorespiratory fitness, HRV, Physical activity, Recovery, Working hours, Work stress

## Abstract

**Background:**

The purpose of this study was to investigate how physical activity (PA), cardiorespiratory fitness (CRF), and body composition are associated with heart rate variability (HRV)-based indicators of stress and recovery on workdays. Additionally, we evaluated the association of objectively measured stress with self-reported burnout symptoms.

**Methods:**

Participants of this cross-sectional study were 81 healthy males (age range 26–40 y). Stress and recovery on workdays were measured objectively based on HRV recordings. CRF and anthropometry were assessed in laboratory conditions. The level of PA was based on a detailed PA interview (MET index [MET-h/d]) and self-reported activity class.

**Results:**

PA, CRF, and body composition were significantly associated with levels of stress and recovery on workdays. MET index (*P* < 0.001), activity class (*P* = 0.001), and CRF (*P* = 0.019) were negatively associated with stress during working hours whereas body fat percentage (*P* = 0.005) was positively associated. Overall, 27.5% of the variance of total stress on workdays (*P* = 0.001) was accounted for by PA, CRF, and body composition. Body fat percentage and body mass index were negatively associated with night-time recovery whereas CRF was positively associated. Objective work stress was associated (*P* = 0.003) with subjective burnout symptoms.

**Conclusions:**

PA, CRF, and body composition are associated with HRV-based stress and recovery levels, which needs to be taken into account in the measurement, prevention, and treatment of work-related stress. The HRV-based method used to determine work-related stress and recovery was associated with self-reported burnout symptoms, but more research on the clinical importance of the methodology is needed.

## Background

Physical activity (PA) is one of the factors that protects against stress [1]. Exercise and psychological stress have similar acute physiological effects because both result in potent increases in cardiovascular, sympathetic, and hypothalamic pituitary adrenocortical responses and decreases in parasympathetic responses [2]. Repeated bouts of exercise lead to physiological adaptations, including decreased resting heart rate (HR) and blood pressure and increased parasympathetic activity [3]. The theory of “cross-stressor adaptation” suggests that regular PA and good fitness lead to adaptations in response to both exercise and psychological stressors [2,4].

The assessment of heart rate variability (HRV) has gained importance as a technique to explore the function of the autonomic nervous system (ANS) and has been widely used to diagnose both psychological and physiological disorders [5]. HRV is the beat-to-beat variation in time of consecutive heartbeats expressed in normal sinus rhythm on an electrocardiogram [6,7]. The separate rhythmic contributions from sympathetic and parasympathetic autonomic activity modulate the heart’s R wave to R wave intervals (RR intervals) of the QRS complex at distinct frequencies [8]. Both sympathetic and parasympathetic activity have been suggested to be associated with the low frequency (LF) range (0.04–0.15 Hz) because parasympathetic activity is a major contributor to the high frequency (HF) range (0.15–0.4 Hz) component in modulation of frequencies of the HR [9,10]. Higher HRV characterizes a healthy person with efficient autonomic mechanisms and good adaptation ability while lower HRV is an indicator of abnormal and insufficient adaptation of the ANS [7,11]. Reduced HRV is suggested to be associated with harmful events in health [12].

A greater amount of reported stress is suggested to be associated with lower HRV [13]. Also, the effects of PA, cardiorespiratory fitness (CRF), and adiposity on HRV profile have been of interest. Chronic exercise increases HRV [14-16], and favorable HRV profiles are associated with higher levels of PA [17] and better CRF [18]. Additionally, obesity has been associated with altered ANS activity [19]. Some studies have found HRV profiles to be relatively poor among obese subjects [20,21] and to improve with weight loss [22].

Work-related stress and physical inactivity are major concerns in society today. The current interest is in reducing both work-related stress and inactivity, and objective methods, such as the HRV-based approach, are needed to diagnose stress symptoms. To make recording of HRV a practical tool for field measurements, new approaches for HRV analysis are needed. For instance, age is an important determinant of HRV, which also has a large inter-individual variability [9,23]. Firstbeat Technologies Ltd. has been developing this kind of diagnostic tool to measure and define stress and recovery based on individual values for HR and HRV (for more information, see the company’s web page [24]). Some stress and recovery variables, calculated with this tool, are suggested to be related to chronic work stress and emotions at work [25]. Using this tool, we investigated how different indicators of PA, CRF, and body composition, which are related to each other, are associated with HRV-based indicators of stress and recovery on workdays among healthy young men. The secondary aim was to evaluate the association between objectively measured stress and self-reported burnout symptoms.

## Methods

### Subjects

The population of this cross-sectional study consisted of groups from two separate studies, the FitFatTwin study (FFT study) and the Body & Future Health study (BFH study). The identical measurements of the cross-sectional FFT study and the baseline measurements of the BHF were pooled for this analysis. In total, 46 men (23 monozygotic twin pairs) participated in the FFT study. The BFH study included 37 men, two of whom were excluded from this study because their HRV recordings included only days off from work. Altogether, 81 healthy males (age range 26–40 y) who were not on regular medication were included in the present study. The twin study was enriched with pairs having within-pair differences in their PA habits. Because the BFH study was originally planned to investigate overweight and physically inactive individuals, it involved the following inclusion criteria: BMI 25.0–35.0 kg/m^2^, waist circumference ≥94 cm, no vigorous exercise (>20 min/session) more than twice a week, and no smoking; for more information, see the registered controlled trial protocol [26]. Levels of HRV-based stress and recovery did not differ between the participants of these two studies. However, the participants from the FFT study had more favorable body composition, better CRF, and a higher self-rated activity class. The characteristics of the participants of the present study are presented in Table 1.

**Table 1:** Characteristics of the study population

	**Mean**	**Range**
Age (y)	34	26–40
Weight (kg)	83.5	51.3–123.2
Height (cm)	179.1	156.5–198.0
BMI (kg/[m]^2^)	26.0	19.8–35.0
Body fat%	24.5	7.6–41.8
VO_2max_^a^ (ml/kg/min)	39.6	23.3–72.6
MET index^b^	3.5	0.1–27.7
Activity class (0–10)	4.4	0.0–9.5
Working time (h)	8.4	2.8–16.0
Sleeping time (h)	7.7	4.7–10.3
Bergen burnout inventory total scores^c^	36	16–74
*Firstbeat variables*		
Work stress (%)	72.8	14.5–99.2
Total stress (%)	49.9	12.6–79.1
Stress index	122.3	80.5–193.3
RMSSD (sleep)	56.78	22.6–150.6
Stress balance (−1 to 1)	0.69	−0.43–1.00
Total recovery (%)	29.3	4.6–67.0
Recovery index (24 h)	92.2	74.0–112.2
Recovery index (sleep)	111.4	45.8–246.9

(n = 81).

^a^n = 78 ^b^MET-h/day, based on retrospective PA interview ^c^n = 80.

Both studies were conducted according to the ethical rules stated in the Declaration of Helsinki. All participants were informed about the study, and they signed written informed consent prior to any measurements. The study protocols were approved by the ethics committee of the Central Finland Health Care District.

### Measurements

The stress and recovery variables were calculated objectively from the HR and HRV recordings, which were executed over three days using Bodyguard, a measurement device developed by Firstbeat Technologies Ltd. (Jyväskylä, Finland). The HRV recordings from these three days were downloaded with Firstbeat Health Software (version 3.1.1.0.). The Firstbeat Health Software first scans the recorded ambulatory RR interval data through an artifact detection filter to perform an initial correction of falsely detected, missed, and premature heart beats [27]. The data were transferred to the Matlab environment (R2007B), where the analyses of physiological variables describing stress and recovery were performed with the Firstbeat Analysis Server program (version 5.3.0.4). This program calculates HRV indices second-by-second using the short-time Fourier transform method and HR- and HRV-derived variables of respiration rate and oxygen consumption using neural network modeling of data [27-29] (for more details, see the white paper by Firstbeat Technologies Ltd. [30]). The program also calculates second-by-second indices of recovery and stress, reflecting activities of the sympathetic (absolute stress vector, ASV) and parasympathetic (absolute recovery vector, ARV) nervous systems. The ASV is calculated from the HR, HF, and LF components and respiratory variables. The ASV is high when HR is elevated, HRV is reduced, and the frequency distribution of HRV is inconsistent because of changes in respiratory period. The ARV, which is calculated from the HR and HF components, is high when HR is close to the basic resting level and HRV is high and regular. The program takes into account individual basic resting HR and HRV values in the determination of the physiological states of the body, including PA, stress state, recovery state, or unrecognized state, and detected based on the above-mentioned variables. The stress and recovery variables used in the present study are based on Firstbeat Analysis Server analyses and are described in Table 2. These variables included the traditional HRV variable RMSSD (root mean square of successive differences), which is a time domain measure of HRV, and plots HRV as the change in normal RR intervals over time [31].

**Table 2:** The description of variables of stress and recovery based on firstbeat analysis server

**Stress variables** For non-exercise data segments, continuous indices of stress are used to identify the time when the body is in a stress state. Stress state is defined as an increased activation in the body when sympathetic nervous system activity is dominating and parasympathetic activation is decreased. Stress can be induced by external and internal stress factors, and the definition of the stress state does not take into account the nature of the stress response, i.e., whether it is positive or negative.	
Work stress	The percentage of stress time during working hours
Total stress	The percentage of stress time in a workday (including leisure time and night)
Stress index	Absolute value characterizing the magnitude of stress processes on a workday (including leisure time and night)
**Recovery variables, 24 hours** Continuous indices of recovery are used to identify the time when the body is in a recovery state. Recovery state is defined as a decreased activation in the body during recovery, rest, and/or peaceful working. This state is related to the lack of external and internal stress factors, and parasympathetic activation is dominating.	
Total recovery	The percentage of recovery time in a workday (including leisure time and night)
Recovery index	Absolute value characterizing the magnitude of recovery processes in a workday (including leisure time and night)
**Recovery variables, sleep time**	
RMSSD	Average of the RMSSD (root mean square of successive differences) vector values during sleep periods. High RMSSD values are associated with increased parasympathetic activity and good recovery while low values during rest indicate poor recovery. The RMSSD value should be over 20 during sleep in a normal situation.
Stress balance	Indicates proportion of time of stress and recovery reactions during sleep periods in the measurement period; the used values are from −1 to 1. Values from 0.5 to 1 indicate good recovery; values from 0 to 0.5 indicate moderate recovery; and values from 0 to −1 indicate weak recovery.
Recovery index	The recovery index gives an estimate of a person’s recovery during sleep time. The 4-h window for determining the recovery index is set to start 30 min after going to bed. The index summarizes several factors, including RMSSD and other HRV variables, and takes into account the other functions of ANS while determining the value for the index.

The HRV data consisted of successfully recorded (RR intervals corrected max 25 percentages) workdays. The stress and recovery values were the mean values of the workdays with the number of days being one (n = 10), two (n = 70), or three (n = 1). The stress and recovery variables were determined from the whole recording day (24 h) or only from working hours or sleeping hours. Working hours, sleeping time, and alcohol consumption were determined from the participants’ measurement diaries, which they were advised to keep over the measurement period. Alcohol consumption was reported daily in standard units of approximately 12 g of ethanol (one unit: 33 centiliter [cl] beer or 12 cl red or white wine or 8 cl fortified wine or 4 cl liquor).

The Bergen Burnout Inventory (BBI) was used in the assessment of occupational burnout. This inventory is employed in some Finnish occupational health services to monitor and screen stress levels [32] and includes 15 questions concerning three subcategories of burnout: exhaustive fatigue, cynicism, and impaired occupational self-respect. Answers to the 15 questions were given using a Likert-type response scale, which was scored from 1 (totally disagree) to 6 (totally agree), and the total scores were between 15 and 90 [33].

Body weight and height were measured in the morning. The body mass index (BMI) is computed as weight/height^2^ (kg/m^2^). The whole body fat percentage was evaluated after fasting 10–12 h using dual-energy X-ray absorptiometry (GE Lunar Prodigy Advance, GE Healthcare). The software used for picture handling and analysis was enCORE™ 2009, version 13.20 (GE Healthcare).

The volume of PA (MET index) was retrospectively inferred using a modified version [34] of the Kuopio Ischemic Heart Disease Risk Factor Study Questionnaire [35], which included questions on leisure-time PA and PA during journeys to and from work, as well as daily activities such as gardening and berry picking. Monthly frequency, mean duration, and mean intensity of each form of activity were evaluated. A multiple of the resting metabolic rate (MET) was assigned for each activity to describe the intensity of the form of PA. The 3-month (BFH study) and 12-month (FFT study) MET indexes for each form of PA were calculated by multiplying the intensity (MET), duration (h), and monthly frequency of the activity, and the MET index was expressed as the sum score of different activities (MET-h/d).

The other method used to estimate PA level was self-reported activity class using a 0–10 scale to represent the activity level of the previous 2–3 months. The values from 0 to 7 were modified from Ross and Jackson’s [36] scale. The values from 7.5 to 10 were added by Firstbeat to include more seriously training individuals and athletes in the scale (for more details, see the white paper by Firstbeat Technologies Ltd. [37]).

CRF was assessed using the cycle ergometer test and a slightly modified World Health Organization protocol [38] with 2-min stages and 25 W/stage increases in workload. The test was submaximal in the BHF study and maximal with breath-by-breath respiratory gas-exchange analysis in the FFT study. The submaximal cycle ergometer test was ended after the workload during which the tested person achieved the submaximal HR level (85–88% from the maximal HR), which was defined from maximal HR based on the participant’s age (210 – (0.65 × age)). In the FFT study, three people did not participate in the maximal cycle ergometer test, and in the case of seven participants, a submaximal test was performed instead of the maximal test for health or motivational reasons. In the analyses, the values used for the maximal oxygen consumption (VO_2max_) are the results of submaximal workload-based calculations for all participants. VO_2max_ was calculated from the submaximal test values of the maximal cycle ergometer test (n = 36). The correlation between this calculation from submaximal workloads and the value from the maximal oxygen consumption test was significant (*P* < 0.001).

### Statistical analysis

Statistical analyses were performed using Mplus version 7 [39]. The statistical significance was set at *P* ≤ 0.05. The estimator used was maximum likelihood with robust standard errors that are robust against non-normality. The effect of clustered data (twins) was controlled using the type complex definition in Mplus. A few missing values (VO_2max_ n = 3 and BBI n = 1) were inferred to be missing at random. Because the variables under interest are known to be related to each other, structural equation modeling was used to investigate how PA, fitness, and body composition are associated with HRV-based indicators of stress and recovery. In the first stage, estimates of each independent variable (body fat percentage, BMI, VO_2max_, MET index, and activity class) were determined to explain the dependent stress and recovery variables. In the second stage, to investigate how much of the variance of the stress and recovery variables is explained by PA, fitness, and body composition together, a latent factor variable was formed from the MET index, activity class, VO_2max,_ body fat percentage, and BMI. In addition, structural equation modeling was used to investigate the association of the HRV-based stress and recovery variables with the BBI total scores.

The model fit was evaluated using the *χ*^2^ test, comparative fit index (CFI), Tucker Lewis Index (TLI), root mean square error of approximation (RMSEA), and standardized root mean square residual (SRMR). For a good-fitting model, the *χ*^2^ test is non-significant; CFI and TLI are at least 0.95; RMSEA is no more than 0.06; and SRMR no more than 0.08 [40].

## Results

Body fat percentage was positively associated (β = 0.306, *P* = 0.005) whereas VO_2max_ (β *= −*0.311, *P* = 0.019), MET index (β = −0.304, *P* < 0.001), and activity class (β *= −*0.326, *P* = 0.001) were negatively associated with HRV-based work stress. Similarly to works stress, the other HRV-based stress variables were associated with body composition, CRF, and PA. However, BMI was positively associated with total stress (β = 0.272, *P* = 0.003) and stress index (β = 0.478, *P* < 0.001). MET index was not associated with stress index (*P* = 0.440).

The indicators of recovery during night-time sleep (RMSSD, stress balance, recovery index) were negatively associated with body fat percentage and BMI and positively associated with VO_2max_. Activity class was positively associated with stress balance value (β = 0.237, *P* = 0.025).

Body fat percentage (β = −0.445, *P* < 0.001) and BMI (β = −0.355, *P* < 0.001) were negatively associated whereas VO_2max_ (β = 0.465, *P* < 0.001), MET index (β = 0.356, *P* < 0.001), and activity class (β = 0.418, *P* < 0.001) were positively associated with total recovery. Body fat percentage (β = −0.343, *P* = 0.002) and BMI (β = −0.309, *P* = 0.001) were negatively associated whereas VO_2max_ was positively associated with 24-h recovery index (β = 0.331, *P* = 0.010). MET index (*P* = 0.414) and activity class (*P* = 0.151) were not associated with 24-h recovery index.

Because the age range of the participants was relatively small and adjustment for age changed the results very minimally, the age-adjusted results are not shown. Adjustments for alcohol consumption, working time, or sleeping time influenced the results only minimally or modestly (Table 3). Adjustments for alcohol most influenced the associations of VO_2max_ with the stress and recovery variables. The results are presented in greater detail in Table 3.

**Table 3:** The association of body composition, cardiorespiratory fitness, and physical activity with stress and recovery

	**No adjustments**			**Alcohol adjusted**			**Working time adjusted**			**Sleeping time adjusted**		
	**β**	**S.E.**	***P***	**β**	**S.E.**	***P***	**β**	**S.E.**	***P***	**β**	**S.E.**	***P***
**Body fat%**												
WS	0.306	0.108	0.005	0.288	0.114	0.012	0.315	0.114	0.006	0.313	0.115	0.006
TS	0.442	0.101	<0.001	0.405	0.107	<0.001	0.242	0.070	0.001	0.429	0.111	<0.001
SI	0.422	0.115	<0.001	0.395	0.108	<0.001	0.426	0.107	<0.001	0.425	0.107	<0.001
TR	−0.445	0.088	<0.001	−0.409	0.087	<0.001	−0.443	0.090	<0.001	−0.419	0.086	<0.001
RI	−0.343	0.113	0.002	−0.325	0.111	0.003	−0.338	0.103	0.001	−0.330	0.111	0.003
RMSSD	−0.293	0.123	0.017	−0.267	0.115	0.020	−0.285	0.109	0.009	−0.283	0.116	0.015
SB	−0.400	0.083	<0.001	−0.377	0.084	<0.001	−0.408	0.076	<0.001	−0.403	0.077	<0.001
RIS	−0.359	0.130	0.006	−0.328	0.127	0.010	−0.348	0.114	0.002	−0.351	0.125	<0.001
**BMI**												
WS	0.162	0.086	0.060	0.139	0.085	0.101	0.176	0.083	0.033	0.171	0.086	0.047
TS	0.272	0.093	0.003	0.210	0.090	0.020	0.268	0.086	0.002	0.251	0.095	0.009
SI	0.478	0.102	<0.001	0.447	0.105	<0.001	0.458	0.091	<0.001	0.482	0.097	<0.001
TR	−0.355	0.084	<0.001	−0.297	0.078	<0.001	−0.351	0.084	<0.001	−0.309	0.084	<0.001
RI	−0.309	0.093	0.001	−0.287	0.094	0.002	−0.298	0.087	0.001	−0.289	0.092	0.002
RMSSD	−0.304	0.106	0.004	−0.274	0.106	0.009	−0.290	0.097	0.003	−0.289	0.107	0.007
SB	−0.379	0.095	<0.001	−0.325	0.101	0.001	−0.395	0.087	<0.001	−0.384	0.092	<0.001
RIS	−0.395	0.102	<0.001	−0.358	0.105	0.001	−0.373	0.095	<0.001	−0.382	0.104	<0.001
**VO**_**2max**_												
WS	−0.311	0.132	0.019	−0.282	0.147	0.055	−0.316	0.147	0.031	−0.317	0.145	0.029
TS	−0.416	0.126	0.001	−0.349	0.155	0.025	−0.411	0.145	0.004	−0.396	0.150	0.008
SI	−0.379	0.096	<0.001	−0.334	0.098	0.001	−0.377	0.094	<0.001	−0.375	0.095	<0.001
TR	0.465	0.111	<0.001	0.405	0.122	0.001	0.469	0.110	<0.001	0.429	0.107	<0.001
RI	0.331	0.128	0.01	0.302	0.139	0.030	0.319	0.133	0.016	0.307	0.143	0.032
RMSSD	0.262	0.125	0.037	0.222	0.125	0.076	0.247	0.123	0.045	0.242	0.130	0.062
SB	0.270	0.073	<0.001	0.145	0.091	0.113	0.283	0.073	<0.001	0.274	0.072	<0.001
RIS	0.294	0.125	0.019	0.243	0.131	0.063	0.275	0.121	0.023	0.276	0.136	0.042
**MET index**												
WS	−0.304	0.113	<0.001	−0.287	0.087	0.001	−0.308	0.087	<0.001	−0.311	0.086	<0.001
TS	−0.385	0.145	0.008	−0.349	0.084	<0.001	−0.384	0.083	<0.001	−0.368	0.086	<0.001
SI	−0.096	0.124	0.440	−0.065	0.105	0.535	−0.098	0.107	0.360	−0.099	0.105	0.343
TR	0.356	0.131	<0.001	0.316	0.102	0.002	0.355	0.095	<0.001	0.321	0.097	0.001
RI	0.138	0.173	0.414	0.120	0.140	0.390	0.135	0.136	0.319	0.122	0.141	0.389
RMSSD	0.058	0.165	0.723	0.032	0.152	0.834	0.054	0.147	0.713	0.046	0.151	0.761
SB	0.097	0.089	0.255	−0.017	0.081	0.835	0.102	0.074	0.172	0.101	0.069	0.141
RIS	0.074	0.173	0.670	0.038	0.159	0.810	0.067	0.150	0.654	0.063	0.158	0.690
**Activity class**												
WS	−0.326	0.102	0.001	−0.298	0.104	0.004	−0.330	0.107	0.002	−0.331	0.109	0.002
TS	−0.434	0.096	<0.001	−0.362	0.102	<0.001	−0.433	0.105	<0.001	−0.424	0.108	<0.001
SI	−0.222	0.113	0.048	−0.175	0.115	0.128	−0.224	0.115	0.051	−0.225	0.114	0.049
TR	0.418	0.097	<0.001	0.336	0.090	<0.001	0.417	0.098	<0.001	0.400	0.101	<0.001
RI	0.175	0.122	0.151	0.146	0.119	0.221	0.172	0.121	0.154	0.164	0.126	0.191
RMSSD	0.090	0.120	0.453	0.047	0.116	0.684	0.086	0.116	0.459	0.082	0.112	0.505
SB	0.237	0.106	0.025	0.082	0.095	0.390	0.241	0.107	0.024	0.239	0.106	0.024
RIS	0.132	0.129	0.305	0.076	0.125	0.544	0.127	0.125	0.311	0.125	0.132	0.343

Standardized estimate (β), standard error (S.E.), and *P* value for body fat%, BMI, VO_2max_, MET index, and activity class in relation to Firstbeat stress and recovery variables (work stress [WS], total stress [TS], stress index [SI], total recovery [TR], recovery index 24 h [RI], RMSSD, stress balance [SB], and recovery index sleep [RIS]).

**Figure 1:**
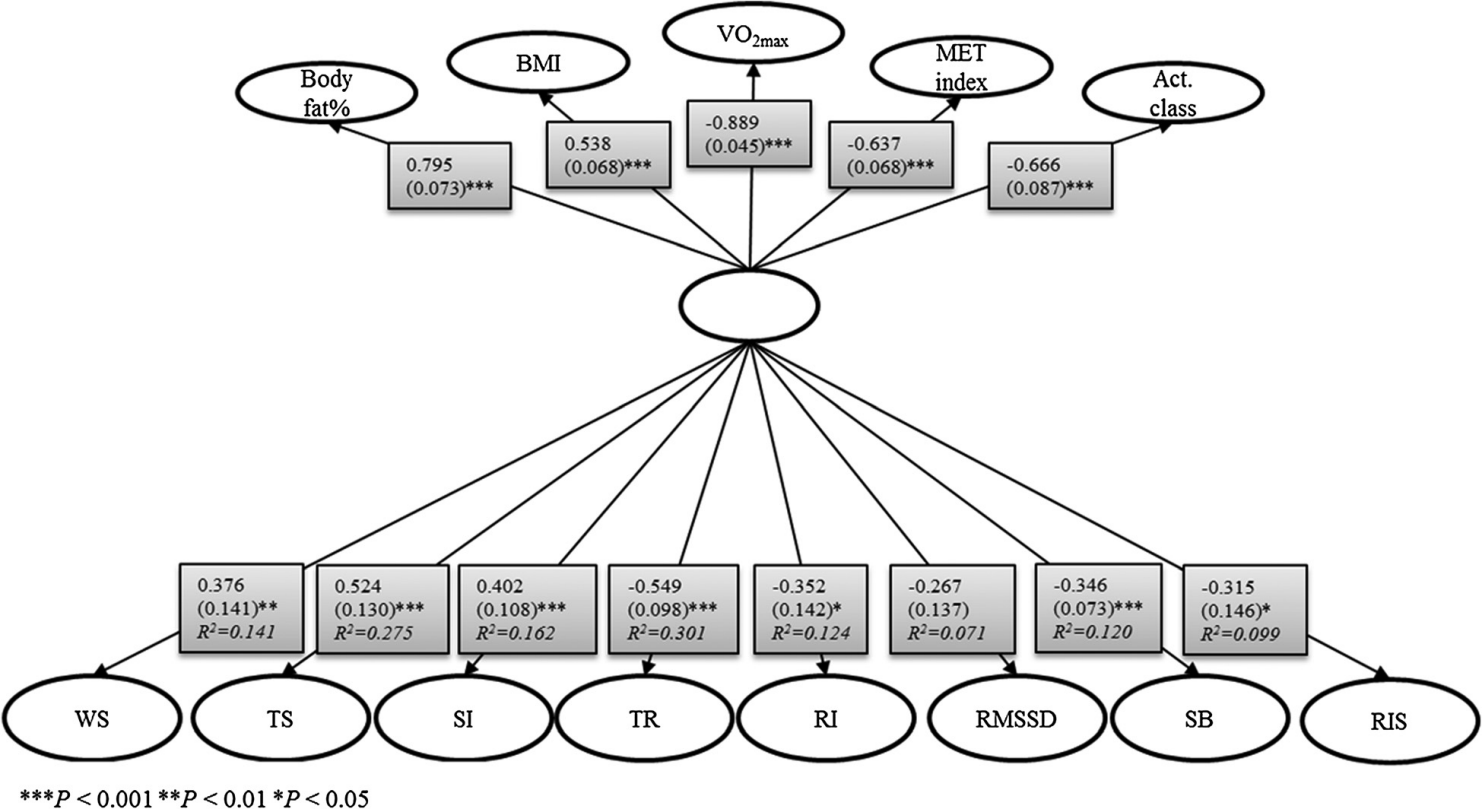
**The variance of stress and recovery accounted for by physical activity, cardiorespiratory fitness, and body composition.** The factor loadings (standardized estimate and S.E.) of body fat%, BMI, VO_2max_, MET index, activity class, and the factor loadings (including *R*^2^) for Firstbeat variables: work stress (WS), total stress (TS), stress index (SI), total recovery (TR), recovery index 24 h (RI), RMSSD, stress balance (SB), and recovery index sleep (RIS).

A latent factor variable (Figure 1) was formed from body fat percentage, BMI, VO_2max_, MET index, and activity class because these variables correlated significantly. The latent variable was a significant explanatory variable for all of the stress and recovery variables except RMSSD. It explained total recovery best at 30.1% of its variance. Among stress variables, the variance of total stress was best explained (27.5%) by the latent factor, which also explained 14.1% of the variance of work stress. The estimated model fit to the data well (*χ*^2^ (35) = 41.09, *P* = 0.22, CFI = 0.99, TLI = 0.98, RMSEA = 0.046, SRMR = 0.06), and all the modification indices were lower than 4, which indicated that were no additional parameters that would have increased the fit of the model for the analysis.

In addition, objectively and subjectively measured stress levels were significantly associated (Table 4). HRV-based work stress (β = 0.272, *P* = 0.003) and total stress (β = 0.304, *P* = 0.001) explained the variation in total score of the BBI questionnaire. Adjustment for age changed the results only minimally, but after adjustment for body fat percentage, associations between work stress and the BBI total score did not persist.

**Table 4:** Stress and recovery in relation to the Bergen Burnout Inventory total scores

	**β**	**S.E.**	***P***
Work stress	0.272	0.091	0.003
Total stress	0.304	0.095	0.001
Stress index	−0.080	0.118	0.500
Total recovery	−0.170	0.092	0.065
Recovery index (24 h)	−0.036	0.109	0.741
RMSSD	−0.009	0.101	0.925
Stress balance	−0.006	0.092	0.947
Recovery index (sleep)	−0.014	0.112	0.898

Standardized estimate (β), standard error (S.E.) and *P*-value.

## Discussion

The results of this study showed that there were significant associations among PA, CRF, body composition, and novel HRV-based levels of stress and recovery in real life on workdays. Greater PA level, better CRF, and more favorable body composition were associated with lower stress levels during working hours as well as with lower stress levels and higher recovery levels throughout the whole day. Better recovery at night was associated with better CRF and a more favorable body composition. Additionally, the relationship between objectively measured stress and self-reported burnout symptoms was significant, and further analysis showed that body composition explained it in part.

The results of the present study are mainly in line with those of earlier studies that used traditional HRV measures. Favorable HRV profiles may be related to PA and CRF [15,16] while stress appears to be linked with reduced leisure-time PA [41] and lower physical fitness [2,42]. Earlier studies have proposed that fit individuals would show attenuated physiological reactivity to psychological stressors compared with sedentary individuals [43,44]. A more recent meta-analytic review by Forcier et al. [2] also provided support for the cross-adaptation hypothesis and suggested that fitness training may increase the ability of cardiovascular systems to control responses to acute stressors and also speed cardiovascular recovery from stress.

The present study found that among men, unfavorable body composition was associated with higher objective stress and lower recovery. According to previous studies that have examined stress as a subjective experience, the association between mental stress and body composition has been inconsistent because evidence both supports [45-49] and refutes [50-53] it. Additionally, the results suggested that body fat percentage explained part of the association between objectively and subjectively measured levels of stress, indicating that body fat percentage affects both physiological stress and subjective experience of stress among men. However, a previous study by Nyberg et al. [46] found that the relationship between stress and unfavorable body composition may not be linear. In an analysis of pooled European data (n = 161,746), both weight gain and weight loss were associated with the onset of job strain so that the cross-sectional association between job strain and BMI formed a ‘U’-shaped curve [46].

The results showed that PA level was not associated with night-time recovery, although regular PA has consistently been associated with better sleep [54]. However, previous findings indicate that PA in the evening may delay the beginning of recovery. Physical fitness may have a role in recovery of HR and HRV after exercise, as could be seen even between trained and highly trained subjects, so that the recovery takes 60–90 min longer in trained subjects [55]. Recovery of HRV also depends on type, intensity, and duration of exercise [56]. In the study of Myllymäki et al. [57], a late-night exercise caused higher HR during the first three sleeping hours of an exercise day compared to a control day. Mischler et al. [58] reported that prolonged exercise had similar effects on HR during sleep. In addition to these effects of exercise on HR, HRV might decrease during the activity and, for instance, high-intensity exercises reduce HRV for 30–60 min afterward in trained and highly trained subjects [55,56]. Hynynen et al. [59] found that both moderate and heavy endurance exercise even in the daytime has an increasing effect on HR and decreasing effect on HRV during sleep in a dose–response manner.

Subjective methods were used to assess the level of PA. The MET index was based on a detailed retrospective interview. This index correlated significantly with measured VO_2max_ values, supporting its validity to assess the level of PA. Subjective assessment may include some reporting bias; however, although the method undertaken was not objective, it had the advantage of taking several months of subjective data into account. The other method was self-reported activity class, which is regularly reported before the HRV measurement by Bodyguard.

One limitation of the present study is that the effect of different intensities of PA was not investigated; however, both light and moderate to vigorous leisure-time activity are related to lower likelihood of burnout [41]. Another limitation is that fat distribution was not taken into account, even though, according to earlier studies, it may have a role in relation to the HRV profile [19,60]. The nature of work in today’s society is rather complex, including inexact and variable working hours and a lack of precise division between work time and leisure time, which causes difficulties in determining the level of HRV-based stress during exact working hours.

The current results cannot be generalized to women because the study population consisted of both physically inactive and active young male employees, who were not heavy drinkers or on regular medication. This population was ideal for this study because alcohol intake, diseases, and medications influence ANS [8]. The effect of possible alcohol intake on HRV was taken into account in the analyses. The fact that our study population consisted of young employees who are still establishing their careers highlights the importance of the findings.

Stress can be explained through several biological processes, such as the inflammatory, dopaminergic, and neuroendocrine systems [61]. However, in the present study, the measurement method for stress and recovery was based on HR and HRV, which are affected by the ANS. Stressful situations result in accentuated sympathovagal antagonism, which may be explained by the interaction of acetylcholine and norepinephrine; consequently, HR may become remarkably unstable [62]. The findings of Tulppo et al. [62] suggest that traditional measures of HRV are not specific for measurement of accentuated sympathovagal interaction. The Firstbeat Analysis Server program connects information from these traditional measures of HRV, HR, and respiratory variables in the determination of stress and recovery states from individual HR and HRV values, but it does not separate eustress from distress.

## Conclusions

This study used special HR- and HRV-based indicators of stress and recovery and found that PA, CRF, and body composition were associated with these indicators on workdays. On one hand, detailed interview-based MET index and self-reported activity class, and on the other hand, body fat percentage and BMI, associated rather similarly with the indicators of stress and recovery. This suggests that easily collected self-reported activity class and BMI can be used as indicators of PA and body composition in clinical work. The current findings support the usability of the objective indicators of stress (work stress and total stress) as they were associated with self-reported occupational burnout symptoms. Overall, the results support the usability of this HRV-based method in the evaluation of stress and recovery in line with some previous findings supporting its validity and reliability [25,63].

## Competing interests

Rusko H. is a stockowner in Firstbeat Technologies Ltd. He did not contribute to writing the conclusions of the study. The other authors declare that they have no competing interests.

## Authors’ contributions

Study conception and design: TT, SM, AT, MR, JK, MK, UK. Acquisition of data: TT, SM, MR, TL, UK. Analysis and interpretation of data: TT, SM, AT, MR, TL, JK, MK, HR, UK. Statistical analysis: TT, AT. Drafting the manuscript: TT. Critical revision of the manuscript for important intellectual content: TT, SM, AT, MR, TL, JK, MK, HR, UK. Final approval of the version to be published: TT, SM, AT, MR, TL, JK, MK, HR, UK. Obtained funding: TT, JK, MK, UK. Administrative, technical, or material support: MK, UK.
